# Supplementary material for: Sodium regulates PLC and IP3R‐mediated calcium signaling in invasive breast cancer cells
Source: Physiol Rep. 2023 Apr 5;11(7):e15663. doi: 10.14814/phy2.15663 (PMC10074044; doi:10.14814/phy2.15663)
Supplement: Supplementary file 1 — Supplementary Figure S1. [file PHY2-11-e15663-s001.docx]

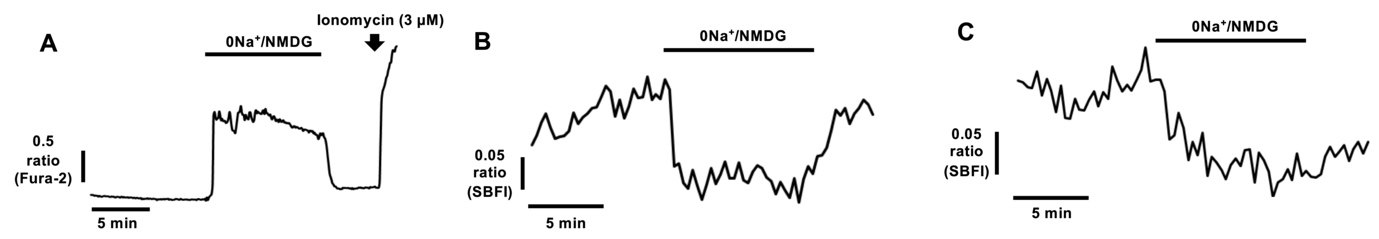


**Supplementary Figure 1: Na^+^-free conditions induce a [Ca^2+^]_i_ rise in MDA-MB-468 cells and deplete [Na^+^]_i_ in MCF-7 and SKBR3 cells.** Fura-2 AM (4 µM) or SBFI AM (4 µM) fluorescence microscopy was used to measure [Ca^2+^]_i_ and [Na^+^]_i_ in cultured human breast cancer cells. Following perfusion with HEPES-PSS, cells were perfused with Na^+^-free HEPES PSS; extracellular Na^+^ was replaced with equimolar N-methyl-D-glucamine (0Na^+^/NMDG) to maintain osmotic balance. Representative traces show the effects of 0Na^+^/NMDG on [Ca^2+^]_i_ in MDA-MB-468 cells (A) and on [Na^+^]_i_ in MCF-7 (B) and SKBR3 (C) cells.
