# Supplementary material for: Sodium regulates PLC and IP3R‐mediated calcium signaling in invasive breast cancer cells
Source: Physiol Rep. 2023 Apr 5;11(7):e15663. doi: 10.14814/phy2.15663 (PMC10074044; doi:10.14814/phy2.15663)
Supplement: Supplementary file 2 — Supplementary Figure S2. [file PHY2-11-e15663-s002.docx]

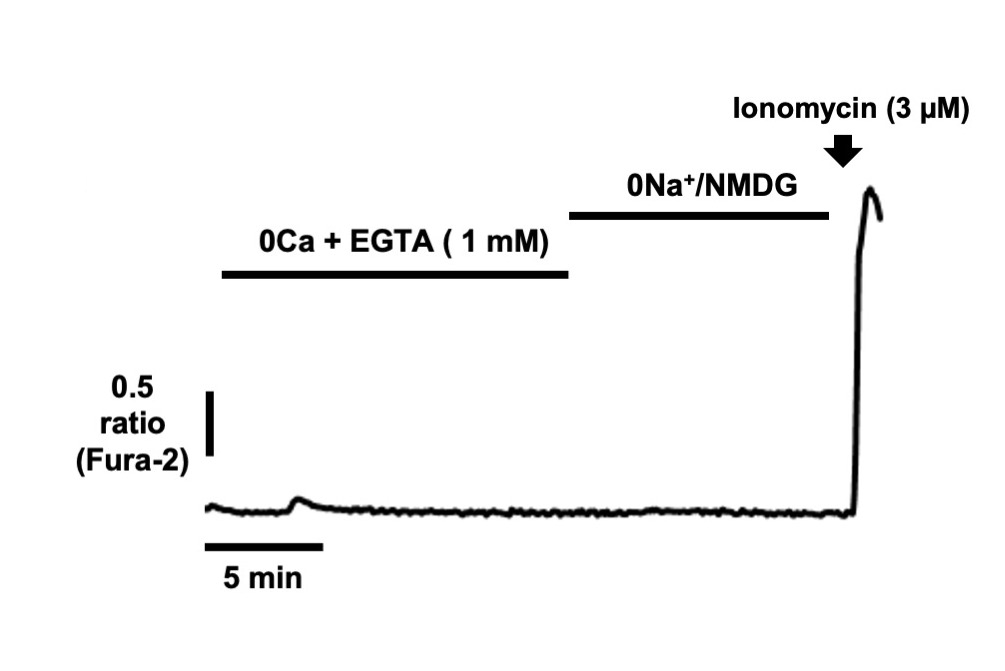


**Supplementary Figure 2: EGTA inhibits [Ca^2+^]_i_ transients induced by removal of extracellular Na^+^.** MDA-MB-231 cells were loaded with fura-2 AM (4 µM) and Ca^2+^ imaging performed during removal of extracellular Ca^2+^ in the presence of EGTA (1 mM), followed by replacement of extracellular Na^+^ with equimolar (0Na^+^/NMDG). Ionomycin (3 µM) was applied at the end of an experiment to elicit a [Ca^+^]_i_ increase as a positive control.
